# Multi-scale investigation of the formation and properties of high-grade rutile TiO_2_ from titanium slags using microwave heating

**DOI:** 10.1098/rsos.171858

**Published:** 2018-06-06

**Authors:** Guo Chen, Jing Pu, Jin Chen, Jinhui Peng, C. Srinivasakannan, Rongsheng Ruan

**Affiliations:** 1 Key Laboratory of Resource Clean Conversion in Ethnic Regions of Education Department of Yunnan, Joint Research Centre for International Cross-border Ethnic Regions Biomass Clean Utilization in Yunnan, Yunnan Minzu University, Kunming 650500, People's Republic of China; 2 State Key Laboratory of Vanadium and Titanium Resources Comprehensive Utilization, Pangang Group Research Institute Co., Ltd., Panzhihua 617000, People's Republic of China; 3 State Key Laboratory of Multiphase Complex Systems, Institute of Process Engineering, Chinese Academy of Sciences, Beijing 100190, People's Republic of China; 4 Chemical Engineering Department, The Khalifa University of Science and Technology, The Petroleum Institute, Abu Dhabi, UAE; 5 Center for Biorefining, Bioproducts and Biosystems Engineering Department, University of Minnesota, 1390 Eckles Ave., Saint Paul, MN 55108, USA

**Keywords:** titanium slag, microwave heating, electron microscopy, phase transition, rutile TiO_2_

## Abstract

In this paper, the phase transition of titanium slag under microwave heating observed through electron microscopy was systematically investigated. The phase identification and transformation as well as the morphology of samples before and after each treatment were characterized using X-ray powder diffraction (XRD), Fourier-transform infrared spectroscopy, Raman spectroscopy and scanning electron microscopy (SEM). The XRD confirmed that sodium salt roasting could modify the phase composition of titanium slag. The microwave roasting in the presence of sodium salt increased the rutile TiO_2_ with high crystallinity after acid leaching. The Raman spectroscopy demonstrated the phase transition of titanium slag from anosovite to rutile TiO_2_ after a series of treatments, and the SEM analyses showed that the surface of calcined products grew plenty of rutile TiO_2_ with typical characteristics. The results indicate a successful process for an effective and efficient way for the utilization of both titanium slag and preparation of rutile TiO_2_.

## 1. Introduction

Titanium dioxide is considered to be one of the best white pigments in the world and is also an important fine chemical. It is extensively used in coating materials, plastics, papers, chemical fibres, rubbers and cosmetic products that are highly consumed in the coating industry [1–3]. The main processes for the production of titanium dioxide are currently either the sulfate or the chlorination process [4]. In the production of titanium dioxide pigments by the sulfate process, the main drawback is the large amount of waste emissions and hence the industry gradually adopted the chlorination process [5,6]. Titanium tetrachloride (TiCl_4_) was first produced through the chlorination of titanium concentrates, and then re-oxidized to titanium dioxide pigment products [7]. This process demands high TiO_2_ content feedstock and the content of contaminants MgO + CaO is limited to less than 1.5% to maximize the recycling of chlorine [8–10].

Recently, TiO_2_ was produced using the industry standard for application in the coating of electric welding rod, which can also be used to make non-conductor. It is especially advantageous for a slag-making agent, with excellent deoxidization effect, and reduces SO_2_ consumption during the manufacturing process to make the surface smooth. Consequently, the increasing use of the process for producing electric welding rod has motivated a search for a more abundant and cheaper raw material than the currently used rutile resources. Therefore, artificial rutile, also known as synthetic rutile, is titanium concentrate consisting mostly of iron and other impurities [11]. The artificial rutile is a substitute for natural rutile as it is similar in the composition and structure [12]. At present, most artificial rutiles are used as the raw materials in the production process of coating welding materials, due to a lack of natural rutile [13]. Also, synthetic rutile is one of the raw materials used in special welding materials.

Microwaves are electromagnetic waves that have a frequency range from around 0.3 GHz to 300 GHz with corresponding wavelengths ranging from 1 m to 1 mm [14,15]. Microwave irradiation technology is widely used in the metallurgy and materials industry, as its heating is uniform, i.e. the material can be heated inside and outside at the same time [16–19].

Although many researchers have investigated the roasting of titanium slag from different sources for the synthesis of rutile TiO_2_, utilization of titanium slag specific to welding material application by roasting has not been reported. In this study, the rutile TiO_2_ was prepared from titanium slags using a new process, which consists of five fundamental steps as follows: alkali leaching, microwave sodium salt roasting, acid leaching, water washing and microwave calcining. The phase identification and transformation as well as the morphology of the products in each step were mainly analysed by X-ray diffraction (XRD), Fourier-transform infrared (FT-IR) spectroscopic analysis, Raman spectroscopy techniques and scanning electron microscopy (SEM). In addition, raw materials used for the preparation of special welding materials need to adhere to the standards regarding the contents of S, P and C.

## 2. Experimental

### 2.1. Materials

The titanium slag used in the present work was received from Kunming (Yunnan province, China). All the chemical reagents were of analytical grade and used without further purification. Pure deionized water was used throughout the preparation of all aqueous solutions. The chemical compositions and the size ranges of titanium slag are listed in tables 1 and 2, respectively. The titanium slag was analysed for element contents and size ranges in accordance with the National Standard of the People's Republic of China (GB 2590.6-81, GB/T 21524-2008). It can be seen from table 1 that the main component of titanium slag is 74.25% of TiO_2._ The titanium slag also contains 2.34% of Ti_2_O_3_, 9.56% of Tree, 3.60% of Al_2_O_3_, 5.68% of SiO_2_, 2.02% of MgO, 0.48% of CaO, and other minor elements such as S, P and C. Table 2 shows the particle distribution of titanium slag is uneven, with 63% of particles in the size range of 96–180 µm, while 37% particles have a size greater than 180 µm.

**Table 1.** Chemical compositions of titanium slag by percentage.

TiO_2_	Ti_2_O_3_	TFe	Al_2_O_3_	SiO_2_	MgO	CaO
74.25	2.34	9.56	3.60	5.68	2.02	0.48

**Table 2.** Size compositions of titanium slag.

size (μm)	>180	150∼180	120∼150	96∼120	75∼96	48∼75	<48
content (%)	37.36	19.27	10.56	11.71	6.73	7.24	5.80

### 2.2. Instruments

The powder XRD (Rigaku D/Max 2200 X, Japan) using CuK*α* radiation (*λ* = 1.5418 Å) was employed to identify the crystalline phase of titanium slag before and after microwave sodium salt roasting. The voltage and anode currents were 35 kV and 20 mA, respectively. Fourier transform infrared spectra were recorded between 4000 and 400 cm^–1^ by FT-IR spectrometer (Nicolet 8700, USA) using the KBr pellets method. The angle of incidence of the IR beam was 45° and 100 scans were collected at a resolution of 4 cm^–1^ and then further averaged using OMNIC spectroscopic software. The Raman spectra of the high titanium slag were recorded using Raman (Renishaw Ramanscope System 1000, UK). Backscattered Raman signals were collected through a microscope and holographic notch filters in the spectrum scattering detection region ranged from 100 cm^–1^ to 1000 cm^–1^. The scanning electron microscope (XL30ESEM-TMP, Philips, Holland) was operated at 20 kV in a low vacuum, and the samples used were analysed to assess their microstructure morphology before and after microwave sodium salt roasting.

**Figure 1.**
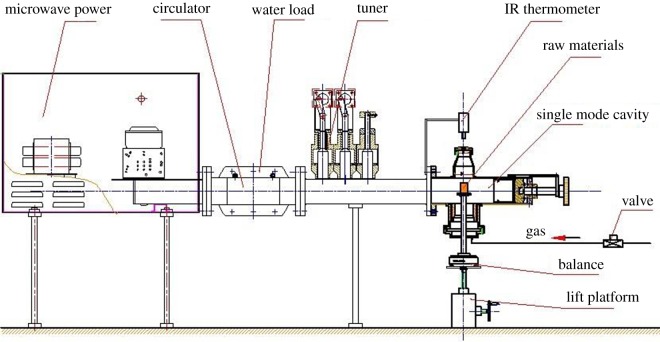
Schematic diagram of microwave heating apparatus.

Experiments were performed in a microwave heating apparatus. The typical microwave heating apparatus consists of a magnetron to produce the microwaves, a waveguide to transport the microwaves, a resonance cavity to manipulate microwaves for a specific purpose, and a control system to regulate the temperature and microwave power. The power supply of the microwave muffle furnace was two magnetrons at 2.45 GHz frequency and 1.5 kW power, which were cooled by water circulation. The inner dimensions of the multi-mode microwave resonance cavity were 260 mm in height, 420 mm in length and 420 mm in width. The magnetrons were cooled using water circulation and the temperature was measured by a Type K thermocouple. The schematic diagram of microwave heating apparatus is shown in figure 1.

### 2.3. Experimental procedures

The titanium slag was dried, ground and sieved to different narrow size fractions. Experiments were performed using a water bath heating system and the microwave heating apparatus using a corundum crucible with a volume of 20 ml. Mixed alkali and titanium slag were homogeneously mixed in a beaker and placed into the water bath. The mixed alkali was prepared by milling NaOH with a mole ratio of 6 : 1, and the mixture was stirred for 60 min at 95°C. The leach residue was mixed with Na_2_CO_3_ at a mass ratio of 4 : 1 (residue : Na_2_CO_3_) and then the mixture was transferred into the corundum crucible. The crucible was placed in the microwave heating apparatus for 120 min at 850°C. The role of sodium salt was to destroy the structure of anosovite under the microwave roasting. After that, the roasted residue was leached by water, and then the filtered solid intermediate was dissolved in the dilute HCl solution for 60 min at 95°C at a liquid–solid mass ratio of 5 : 1. The intermediates were filtered, washed with distilled water and dried at 105°C for 4 h. Finally, the dried products were calcined in a microwave heating apparatus at 950°C for 3 h.

## 3. Results and discussion

**Figure 2.**
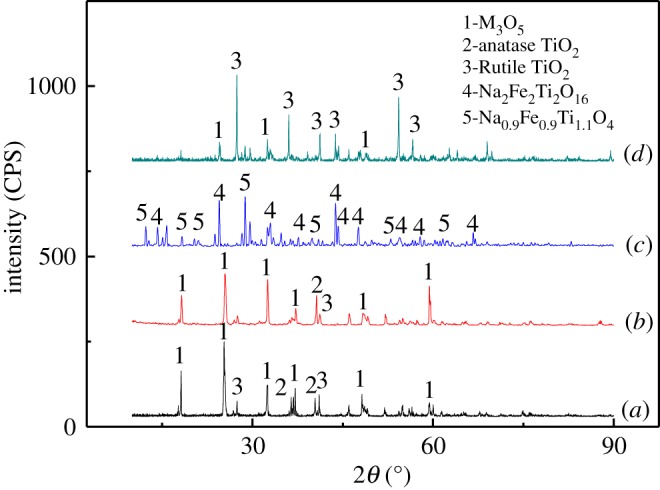
XRD of samples before and after microwave sodium salt roasting: (*a*) raw materials; (*b*) basic leached residue; (*c*) roasted residue; and (*d*) calcined product.

The phase structures of the sample before and after microwave sodium salt roasting were characterized by the XRD technique, and the results are shown in figure 2. It can be seen from figure 2*a* that anosovite (M_3_O_5_) and anatase TiO_2_ (JCPDS card No.89-4203) are the main crystalline compounds of the titanium slag, and M_3_O_5_ is mainly composed of Ti_3_O_5_ (JCPDS card No.82-1137), Ti_2_O_3_ (JCPDS card No.71-1046), Fe_3_Ti_3_O_10_ (JCPDS card No.43-1011), FeTi_2_O_5_ (JCPDS card No.89-8065), MgTi_2_O_5_ (JCPDS card No.89-6945) and other solid-solution minerals [20–22]. The results indicate that the raw materials consist of anosovite, anatase TiO_2_ and a small amount of rutile TiO_2_ (JCPDS card No.71-0650). It can be seen from figure 2*b* that the phase structure has not changed greatly from the original titanium slag, which also consists of anosovite. Alkali leaching was a process primarily aimed at removing impurities, hence no phase transformation is observed. The XRD pattern of the basic leached residue roasted with Na_2_CO_3_ at 850°C is shown in figure 2*c*. The messy and rough diffraction peaks indicate that the sample is mainly composed of titanate. The chemical reactions during the sodium salt roasting can be represented by the following equations:

3.1 $$\begin{array}{r} \begin{array}{rl} {\text{FeTi}}_{2}{\text{O}}_{5}+2{\text{Na}}_{2}{\text{CO}}_{3} & =2{\text{Na}}_{2}{\text{TiO}}_{3}+\text{FeO}+2{\text{CO}}_{2}\\ \Delta {\text{G}}_{T}^{\theta } & =-0.253\text{T}+208.81{\text{kJ mol}}^{-1}, \end{array} \end{array}$$

3.2 $$\begin{array}{r} \begin{array}{rl} {\text{MgTi}}_{2}{\text{O}}_{5}+2{\text{Na}}_{2}{\text{CO}}_{3} & =2{\text{Na}}_{2}{\text{TiO}}_{3}+\text{MgO}+2{\text{CO}}_{2}\\ \Delta {\text{G}}_{T}^{\theta } & =-0.261\text{T}+167.54{\text{kJ mol}}^{-1}, \end{array} \end{array}$$

3.3 $$\begin{array}{r} \begin{array}{rl} {\text{Al}}_{2}{\text{TiO}}_{5}+2{\text{Na}}_{2}{\text{CO}}_{3} & ={\text{Na}}_{2}{\text{TiO}}_{3}+2{\text{NaAlO}}_{2}+2{\text{CO}}_{2}\\ \Delta {\text{G}}_{T}^{\theta } & =-0.231\text{T}+231.15{\text{kJ mol}}^{-1}, \end{array} \end{array}$$

3.4 $$\begin{array}{r} \begin{array}{rl} {\text{FeTiO}}_{3}+{\text{Na}}_{2}{\text{CO}}_{3} & ={\text{Na}}_{2}{\text{TiO}}_{3}+\text{FeO}+{\text{CO}}_{2}\\ \Delta {\text{G}}_{T}^{\theta } & =-0.236\text{T}+199.98{\text{kJ mol}}^{-1} \end{array} \end{array}$$

3.5 $$\begin{array}{r} \begin{array}{rl} {\text{TiO}}_{2}+2{\text{Na}}_{2}{\text{CO}}_{3} & ={\text{Na}}_{2}{\text{TiO}}_{3}+{\text{CO}}_{2}\\ \Delta {\text{G}}_{T}^{\theta } & =-0.127\text{T}+121.59{\text{kJ mol}}^{-1}. \end{array} \end{array}$$

Based on these reaction patterns, a sodium salt roasting temperature higher than 1141.9 K (950°C) was used so as to modify the phase composition of titanium slag. As shown in figure 2*d*, the diffraction peaks of rutile TiO_2_ after calcination are smooth and clear. The strongest preferential orientation of the (110) plane of rutile TiO_2_ appears at 27.42°, and the other preferential orientations of the (110) and (211) planes are observed at 36.08° and 54.28°, respectively. The narrow diffraction peaks also indicate that the main crystalline phase in products is rutile TiO_2_, including little anosovite. It also demonstrates that microwave sodium salt roasting could modify the phase composition of titanium slag. The chemical compositions of rutile TiO_2_ after microwave treatment are presented in table 3. In this study, the samples after roasting were used as raw materials for the production of welding electrodes. The high-quality rutile is defined by contents of S, P and C below 0.030%.

**Table 3.** Chemical compositions of rutile TiO_2_ after microwave treatment (wt%).

composition	TiO_2_	Fe_2_O_3_	Al_2_O_3_	MgO	SiO_2_	CaO	S	P	C
mass (%)	81.323	1.355	0.112	0.353	3.213	1.352	0.022	0.001	0.013

The following equation gives the chemical reaction of prepared rutile TiO_2_ during microwave sodium salt roasting:

3.6 $$\text{N}{\text{a}}_{2}\text{Ti}{\text{O}}_{3}+2{\text{H}}^{+}=\text{Ti}{\text{O}}_{2}+2\text{N}{\text{a}}^{+}+{\text{H}}_{2}\text{O}.$$

**Figure 3.**
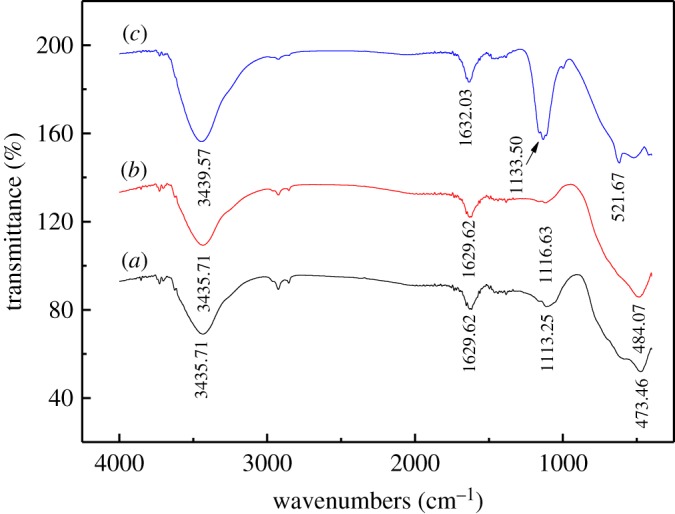
FT-IR spectra of titanium slag: (*a*) raw materials; (*b*) basic leached residue; and (*c*) calcined product.

The surface chemical functional groups of titanium slag were firstly identified by using FT-IR spectrometry (figure 3). Figure 3*a* illustrates the FT-IR spectra of titanium slag with absorption bands at 3435.71 cm^–1^, 1629.62 cm^–1^, 1113.25 cm^–1^ and 473.46 cm^–1^. The strong adsorption bands at 3435.71 cm^–1^ and 473.46 cm^–1^ are due to the O-H stretching vibrations and the stretching vibrations of octahedral metal ions in the TiO_2_ units, respectively. In the region near 1629.62 cm^–1^, only absorbed water-related vibrations are observed, and the absorption band at 1113.25 cm^–1^ can be attributed to bending vibrations of O-H. It can be seen from figure 3*b* that the surface chemical functional groups of basic leached residues and the raw materials are virtually identical, with only a band at 473.46 cm^–1^ individually blueshifted to 484.07 cm^–1^. The FT-IR spectra of the prepared synthetic rutile calcined at 950°C for 60 min is shown in figure 3*c* with absorption bands at 3439.57 cm^–1^, 1632.03 cm^–1^, 1133.50 cm^–1^ and 521.67 cm^–1^. Notably, the band at 473.46 cm^–1^ gradually blueshifted towards high wavenumbers after microwave calcination, which again is the most obvious change compared with the raw materials. It can be attributed to the phase transformation from the anatase into rutile TiO_2_ over that temperature range.

**Figure 4.**
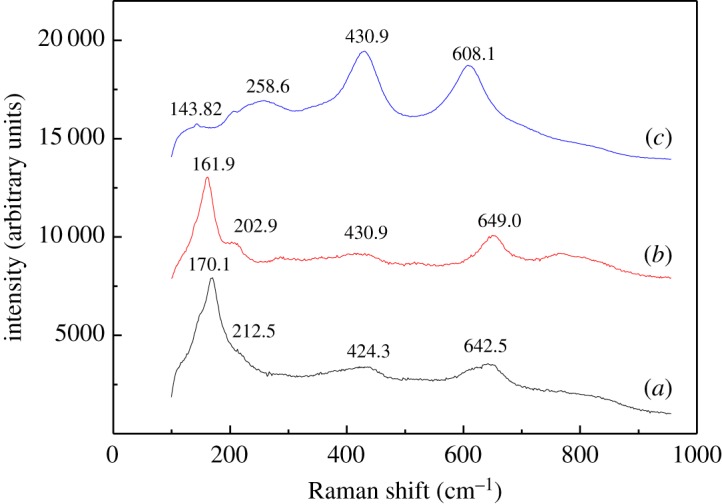
Raman spectra of titanium slag: (*a*) raw materials; (*b*) basic leached residue; and (*c*) calcined product.

In order to compare the phase changes, titanium slag was investigated by Raman spectroscopy and the results are shown in figure 4. The titanium slag sample in figure 4*a* shows four Raman active modes at 170.1 cm^–1^, 212.5 cm^–1^, 424.3 cm^–1^ and 642.5 cm^–1^. The strongest peak at 170.1 cm^–1^ is assigned to the wagging vibrations of O-Ti-O bonds of Ti_3_O_5_, and the frequencies of Raman bands identified at 212.5 cm^–1^ can be attributed to the symmetric stretching vibrations of Ti_2_O_3_. Two weak peaks at 424.3 cm^–1^ and 642.5 cm^–1^ are probably due to rutile TiO_2_ and anatase TiO_2_, respectively, which agrees well with the XRD analysis. As shown in figure 4*b*, the Raman peaks detected at 161.9 cm^–1^, 202.9 cm^–1^, 430.9 cm^–1^ and 649.0 cm^–1^ correspond well with Ti_3_O_5_, Ti_2_O_3_, rutile TiO_2_ and anatase TiO_2_. It can be seen from figure 4*c* that the peaks at 143.8 cm^–1^ and 258.6 cm^–1^ are barely visible, indicating that the anosovite is completely broken after calcination. The most obvious change in the spectra are the appearance of strong peaks at 430.9 cm^–1^ and 608.1 cm^–1^ due to the rutile TiO_2_. The Raman spectroscopy results show the phase transition of titanium slag from anosovite to rutile TiO_2_ after a series of treatments.

**Figure 5.**
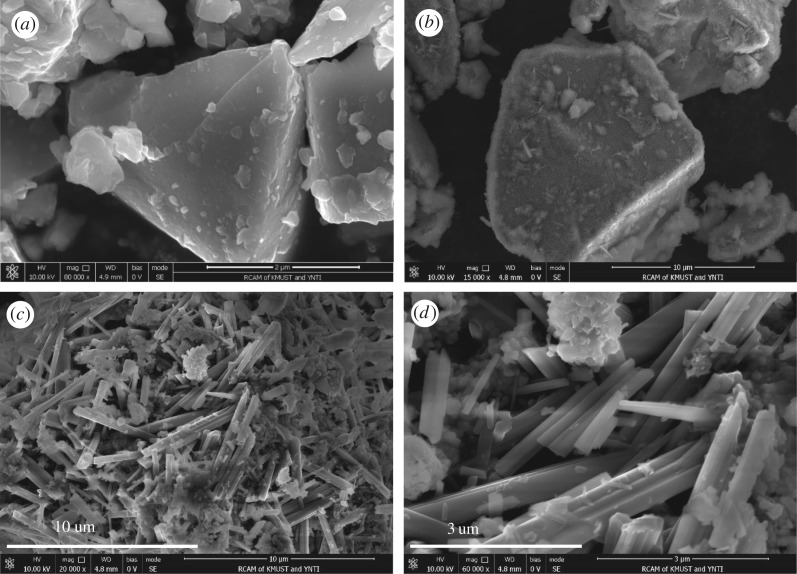
SEM images of titanium slag: (*a*) raw materials; (*b*) basic leached residue; (*c*) and (*d*) calcined product.

Scanning electron microscopy (SEM) was employed to investigate the change of morphology of titanium slag (figure 5). It can be seen from figure 5*a* that the original titanium slag has a denser and smoother surface morphology, with some small particles appearing on the titanium slag surface. As shown in figure 5*b*, the alkali-pretreated sample has few eroded pits and the surface is coarse and poriferous. The eroded pits can be attributed to the process of removing most of the impurities such as Si, Al and Mn with the sodium hydroxide solution. The morphology of the sample after calcination is shown in figure 5*c*,*d*. It can be seen that the surface of the calcined product shows the development of bars and needle-like structures. The bars exhibit a smooth surface, which are the microstructure characteristics of rutile TiO_2_.

## 4. Conclusion

The rutile TiO_2_ was prepared from titanium slag using a new process, which consists of five fundamental steps: alkali leaching, microwave sodium salt roasting, acid leaching, water washing and microwave calcining. The results of XRD showed that anosovite (M_3_O_5_) and anatase TiO_2_ were the main crystalline compounds in titanium slag. The Raman spectroscopy results showed that the wagging vibrations of O-Ti-O bonds of Ti_3_O_5_ at 143.8 cm^–1^ were barely visible, and the peaks at 430.9 cm^–1^ and 608.1 cm^–1^ were attributed to the rutile TiO_2_, which became stronger after calcination. The FT-IR analysis showed that the band at 473.46 cm^–1^ attributed to the stretching vibrations of octahedral metal ions in the TiO_2_ units gradually blueshifted towards a high wave number after microwave calcination. SEM analysis showed that the surface of calcined products grew plenty of rutile TiO_2_ with typical characteristics. The high-quality rutile is defined as such with content of S, P and C below 0.030%. The results indicate a successful process for an effective and efficient way for utilization of both titanium slag and preparation of coating of welding materials for producing electric welding rod.

## Data accessibility

This article does not contain any additional data.
